# Photobiomodulation inhibits the activation of neurotoxic microglia and astrocytes by inhibiting Lcn2/JAK2-STAT3 crosstalk after spinal cord injury in male rats

**DOI:** 10.1186/s12974-021-02312-x

**Published:** 2021-11-05

**Authors:** Xuankang Wang, Xin Li, Xiaoshuang Zuo, Zhuowen Liang, Tan Ding, Kun Li, Yangguang Ma, Penghui Li, Zhijie Zhu, Cheng Ju, Zhihao Zhang, Zhiwen Song, Huilin Quan, Jiawei Zhang, Xueyu Hu, Zhe Wang

**Affiliations:** 1 Department of Orthopedics, Xijing Hospital, Fourth Military Medical University, Xi’an, 710032 Shaanxi China; 2 967 Hospital of People’s Liberation Army Joint Logistic Support Force, Dalian, 116044 Liaoning China

**Keywords:** Neuroinflammation, Spinal cord injury, Microglia, Astrocytes, Lcn2, JAK2-STAT3 pathway

## Abstract

**Background:**

Neurotoxic microglia and astrocytes begin to activate and participate in pathological processes after spinal cord injury (SCI), subsequently causing severe secondary damage and affecting tissue repair. We have previously reported that photobiomodulation (PBM) can promote functional recovery by reducing neuroinflammation after SCI, but little is known about the underlying mechanism. Therefore, we aimed to investigate whether PBM ameliorates neuroinflammation by modulating the activation of microglia and astrocytes after SCI.

**Methods:**

Male Sprague–Dawley rats were randomly divided into three groups: a sham control group, an SCI + vehicle group and an SCI + PBM group. PBM was performed for two consecutive weeks after clip-compression SCI models were established. The activation of neurotoxic microglia and astrocytes, the level of tissue apoptosis, the number of motor neurons and the recovery of motor function were evaluated at different days post-injury (1, 3, 7, 14, and 28 days post-injury, dpi). Lipocalin 2 (Lcn2) and Janus kinase-2 (JAK2)-signal transducer and activator of transcription-3 (STAT3) signaling were regarded as potential targets by which PBM affected neurotoxic microglia and astrocytes. In in vitro experiments, primary microglia and astrocytes were irradiated with PBM and cotreated with cucurbitacin I (a JAK2-STAT3 pathway inhibitor), an adenovirus (shRNA-Lcn2) and recombinant Lcn2 protein.

**Results:**

PBM promoted the recovery of motor function, inhibited the activation of neurotoxic microglia and astrocytes, alleviated neuroinflammation and tissue apoptosis, and increased the number of neurons retained after SCI. The upregulation of Lcn2 and the activation of the JAK2-STAT3 pathway after SCI were suppressed by PBM. In vitro experiments also showed that Lcn2 and JAK2-STAT3 were mutually promoted and that PBM interfered with this interaction, inhibiting the activation of microglia and astrocytes.

**Conclusion:**

Lcn2/JAK2-STAT3 crosstalk is involved in the activation of neurotoxic microglia and astrocytes after SCI, and this process can be suppressed by PBM.

## Introduction

The incidence of spinal cord injury (SCI) has gradually increased in recent years, placing great burdens on individuals, families and society, but there are no effective interventions [1]. Neuroinflammation after SCI is receiving increasing attention due to its complex and contradictory effects. Neuroinflammation is initially a defense mechanism that removes cell debris and promotes tissue repair [2], but persistent inflammation is harmful and may lead to failure of recovery. Microglia and astrocytes, which are innate immune cells in situ, are ubiquitously distributed throughout the brain and spinal cord and play important roles in pathology and repair processes [3]. According to their activated states under different pathological conditions, microglia and astrocytes may secondarily acquire specific reactive phenotypes with different impacts on injury or disease progression. These phenotypes can be divided broadly into neurotoxic and neuroprotective phenotypes (or pro-inflammatory and anti-inflammatory phenotypes) [4]. Neurotoxic microglia express inducible nitric oxide synthase (iNOS) and other inflammatory markers and tend to release destructive mediators, including tumor necrosis factor-α (TNF-α), interleukin (IL)-1β and IL-6 [5], which are harmful to tissue repair. Neurotoxic astrocytes with activation of complement C3 (C3) are considered to have lost most of their normal functions. This functional loss leads to abnormal synthesis and release of neurotransmitters, disrupted synapse formation, and rapid killing of neurons and mature differentiated oligodendrocytes [6]. Notably, microglia and astrocytes are closely related; neurotoxic astrocytes are induced by IL-1α, component 1q (C1q) and TNF-α secreted by activated microglia [6]. Therefore, many studies have regarded microglia and astrocytes as one unit and have focused on exploring the mechanisms of microglial neurotoxicity and astrocyte activation in order to find potential intervention targets [7–9].

Photobiomodulation (PBM), also known as low-level laser therapy (LLLT), refers to the use of low-energy lasers to irradiate tissues at specific wavelengths in order to activate cytochrome c oxidase (CCO), enhance mitochondrial function, and improve blood flow and tissue energy metabolism [10, 11]. As an effective therapy for central nervous system (CNS) injury and disease models, PBM has been used in the treatment of traumatic brain injury (TBI), ischemic stroke, Alzheimer’s disease (AD), Parkinson’s disease (PD), SCI, and other conditions [12–14]. Previous studies by our team have shown that the application of PBM in an SCI mouse model can affect the polarization of bone marrow-derived macrophages (BMDMs), thus inhibiting the formation of glial scars, alleviating inflammation of the injured area, and promoting the recovery of motor function [15, 16]. However, the effect of PBM on neurotoxic microglia and astrocytes has not been elucidated. Therefore, we hypothesized that PBM can inhibit the neurotoxicity of activated microglia and astrocytes, thereby promoting SCI repair.

Lipocalin 2 (Lcn2), also known as neutrophil gelatinase-associated lipocalin (NGAL), is an acute-phase response protein that is upregulated in CNS diseases or injury and plays a regulatory role as an amplifier in neuroinflammation [17–19]. Both in vivo and in vitro studies have shown that Lcn2 is an autocrine mediator of reactive glial cells, which can further promote the activation of microglia and astrocytes and increase the sensitivity of neurons to death signals [20–22]. The Janus kinase-2 (JAK2)-signal transducer and activator of transcription-3 (STAT3) pathway is also activated in CNS injury depending on the degree of severity of the injury; this pathway regulates the expression of various genes that are essential to basic functions, including cell proliferation and differentiation, synaptic plasticity, learning and memory, and immunomodulation [23–25]. There is evidence that inhibiting the activation of the JAK2-STAT3 pathway after SCI can effectively reduce secondary damage and promote repair [26–28]. Therefore, targeting the Lcn2 and JAK2-STAT3 pathways, which play key roles in neuroinflammation after SCI, has been verified as a promising treatment strategy [22, 29]. In this study, we reveal for the first time that Lcn2/JAK2-STAT3 crosstalk plays an important role in the activation of neurotoxic microglia and astrocytes. Our findings suggest that inhibiting this interaction may be a mechanism by which PBM exerts a therapeutic effect to promote SCI repair.

## Materials and methods

### Animals

Male Sprague–Dawley (SD) rats (250–300 g) were supplied by the Animal Centre of Air Force Medical University, Xi’an, Shaanxi Province, People’s Republic of China. The animal experimental protocol was approved by the Animal Protection and Utilization Committee of Air Force Medical University. The rats were housed under standard conditions (temperature: 22–25 °C, relative humidity: 45–65%, light:dark cycle: 12 h:12 h, food and water ad: libitum). A total of 279 subjects were included, and they were randomly divided into three groups: the sham control group (*n* = 94), the SCI + vehicle group (*n* = 94) and the SCI + PBM group (*n* = 91).

### Animal model construction

SCI was established with the modified bilateral spinal cord clamp model we have described previously [15]. Briefly, rats were anaesthetized with an intraperitoneal injection of pentobarbital sodium (50 mg/kg). Midline skin incisions were made, the T10 spinous processes were exposed, and a laminectomy was performed at T10. Compression was conducted by placing forceps (Fine Science Tools) lateral to the exposed spinal cord. Complete closure was performed with modification by adding a metal spacer between the blades to obtain a gap of 0.5 mm for 40 s. The forceps were removed, and the bleeding was stopped. Then, laser fibers were implanted; the detailed procedures are described in our previous report [30]. In brief, the wound was washed with sterile saline to remove blood and debris, and then the front end of the laser fiber was fixed to the soft tissue at the T10 spinous region by using absorbable sutures. The rear end of the laser fiber was interfaced with the laser irradiation device (MW-GX-808, Lei Shi Optoelectronics Co., Ltd.) to confirm that the laser energy projected directly onto the surface of the spinal cord, and then the rear end of the laser fiber was stitched tightly to the skin. After washing the incision, the muscles and skin were sutured carefully layer by layer. Throughout the experiment, the rats were placed on a blanket and maintained at a constant temperature of approximately 37 °C. An antibiotic was given by intraperitoneal injection for 5 days after the operation. Urinary retention was relieved by twice-daily bladder expression until the recovery of spontaneous micturition. The sham-operated rats underwent every surgical step except for spinal cord compression; laser fibers were implanted in both the SCI + vehicle and SCI + PBM groups.

### Laser irradiation

The parameters of laser irradiation were set according to previous studies [15, 31]. Rats were lightly anaesthetized, put into a warm cage, and continuously irradiated with an 810-nm diode laser (150 mW output power). The first irradiation was carried out immediately post surgery, and PBM was continued for 60 min daily beginning at 9 am for two consecutive weeks in the SCI + PBM group. The rats in the sham group and SCI + vehicle group were treated identically except for the laser application.

### Functional assessment

The Basso–Beattie–Bresnahan (BBB) scale and the Louisville Swim Scale (LSS) were used to evaluate locomotor function as previously described [32, 33]. The BBB score was calculated to assess hindlimb locomotor function with a scale ranging from 0 to 21; a score of 0 represented no observable movement, and a score of 21 represented normal movement. The BBB score was evaluated before the operation and at 1 day post-injury (dpi), 3 dpi, 5 dpi, 7 dpi, 14 dpi, 21 dpi and 28 dpi. LSS scoring was conducted to assess the movement and alternation of the hindlimbs. The dependence on the forelimbs, body angle and trunk instability were also evaluated. Rats were trained to swim from one edge of the glass tank to the other for the swimming test before the operation. The LSS score was evaluated before the operation and at 3 dpi, 7 dpi, 14 dpi, and 28 dpi. Each animal was tested twice, and two observers who were blinded to the animal groups evaluated each animal independently (*n* = 6 rats per group).

### Tissue processing

Two methods were used to process spinal cord tissue for the different experiments. In the first method, for immunofluorescence and TUNEL assays, samples were collected separately at 1 dpi, 3 dpi, 7 dpi, 14 dpi and 28 dpi (*n* = 6 rats per group at each time point). The rats were perfused with 4% paraformaldehyde in phosphate buffer (4 °C), and an approximately 2-cm segment containing the same length of normal spinal cord tissue at a symmetrical rostral and caudal direction to the epicenter was carefully dissected from the lesion site. After being placed in 4% paraformaldehyde in phosphate buffer for 4–6 h, the spinal cord was transferred to 25% glucose phosphate buffer at 4 °C for dehydration until it sank. The dehydrated tissue was removed and surface water drained, and the target tissue was smoothed with a scalpel and placed face-up on the specimen chuck. Optimum cutting temperature (OCT) embedding agent was dropped around the tissue, and the specimen chuck was placed on the quick-freeze table of a cryostat (CM1900, Leica) for quick-freezing and embedding. When the OCT became white and hard, it was sectioned. Approximately 10 μm thick serial sagittal sections were sliced and then placed on slides. The sections near the center of each subject sample were selected for storage at − 20 ℃. For subsequent molecular biology experiments, rats were perfused with saline (4 °C), and an approximately 1 cm spinal cord segment was dissected from the lesion site, snap-frozen in liquid nitrogen and stored at − 80 °C. For real-time quantitative PCR (RT-PCR), samples were collected at 1 dpi, 3 dpi, 7 dpi, 14 dpi and 28 dpi (*n* = 5 rats per group at each time point). For Western blotting, samples were collected at 1 dpi, 3 dpi, 7 dpi, 14 dpi and 28 dpi (*n* = 6 rats per group at each time point). For enzyme-linked immunosorbent assay (ELISA), samples were collected at 3 dpi (*n* = 6 rats per group).

### Immunofluorescence

Immunofluorescence was performed to calculate the number of positive cells and to evaluate the activation states of microglia and astrocytes. Sections were rinsed three times in phosphate-buffered saline (PBS) for 5 min each at room temperature and then blocked with 1% donkey serum containing 0.3% Triton X-100 for 30 min. The sections were then incubated with primary antibodies overnight at 4 °C and with appropriate secondary antibodies at 37 °C for 2 h the next day. Finally, to label the nuclei, the sections were counterstained with 4′,6-diamidino-2-phenylindole (DAPI). The following primary antibodies were used: anti-NeuN (1:100, Abcam, ab177487), anti-glial fibrillary acidic protein (GFAP) (1:400, ab4674, Abcam), anti-ionized calcium binding adapter molecule 1 (Iba1) (1:500, Abcam, ab178846), anti-iNOS (1:500, Abcam, ab49999), anti-C3 (1:300, Abcam, ab200999), and anti-Lcn2 (1:200, Abcam, ab63929). Images were obtained under a fluorescence microscope (BX51, Olympus).

### TUNEL assay

To assess the level of tissue apoptosis, a TUNEL kit (Beyotime) was used following the manufacturer's protocol. Frozen sections were washed in PBS for 5 min and then blocked with 1% donkey serum containing 0.3% Triton X-100 for 30 min. TUNEL detection solution was prepared and added to the samples, and the samples were incubated at 37 °C for 2 h in the dark. The nuclei were counterstained with DAPI. Images were obtained under a fluorescence microscope (BX51, Olympus).

### Image analysis and quantification

For each animal, we chose three nonconsecutive sections near the central canal of the spinal cord with intervals of 100 μm. The GFAP-positive cells and Iba1-positive cells were counted in a region of interest (ROI) that was within ± 600 μm of the lesion epicenter in the rostral and caudal directions. The NeuN-positive cells and TUNEL-positive cells were counted in an ROI that was within ± 150 μm of the lesion epicenter in the rostral and caudal directions of the ventral horn of the spinal cord. The cell counting method was followed by calibration with an empirical method to ensure that unbiased data were collected [34, 35]. The cell counts were averaged from five independent fields (randomly selected from within the ROI at a symmetrical rostral and caudal distance from the epicenter) for analysis (*n* = 6 rats per group at each time point). All quantifications were performed by experimenters who were blinded to the experimental groups by using ImageJ software (NIH).

### RT-PCR

Total RNA was extracted from injured spinal cord tissue stored at − 80 ℃ with a commercial RNA extraction kit (Takara Bio) according to the manufacturer’s protocol. RNA integrity was evaluated, and purified RNA with an RNA integrity number (RIN) > 7 was reverse-transcribed. cDNA was reverse-transcribed from 1 μg of RNA template with a PrimeScript RT Master Mix Kit (Takara Bio). Fluorescent RT-PCR was performed in 10 μL of solution containing 1.5 μL of cDNA, 2.5 μL of ddH2O, 1 μL of primer and 5 μL of SYBR Green according to the manufacturer’s instructions. Clean reads were obtained after removing low-quality reads and those containing adapter and poly-N sequences. The GAPDH gene was used as an internal reference. The RT-PCR data were analyzed by the 2^−ΔΔCT^ method. The sequences of the primer pairs for the target genes are provided in Table 1.

**Table 1:** RT-PCR primer sequences

Gene	Forward primer sequence (5′–3′)	Reverse primer sequence (5′–3′)
Lcn2	CCGACACTGACTACGACCAG	AATGCATTGGTCGGTGGGAA
iNOS	TGGTGAGGGGACTGGACTTT	ATCCTGTGTTGTTGGGCTGG
C3	CCAGCTCCCCATTAGCTCTG	GCACTTGCCTCTTTAGGAAGTC
Gfap	AACCGCATCACCATTCCTGT	TCCTTAATGACCTCGCCATCC
Amigo2	GTTCGCCACAACAACATCAC	GTTTCTGCAAGTGGGAGAGC
Serping1	TGGCTCAGAGGCTAACTGGC	GAATCTGAGAAGGCTCTATCCCCA
C1q	TCTGCACTGTACCCGGCTA	CCCTGGTAAATGTGACCCTTTT
IL-1α	GCACCTTACACCTACCAGAGT	AAACTTCTGCCTGACGAGCTT
TNF-α	CCCTCACACTCAGATCATCTTCT	GCTACGACGTGGGCTACAG
IL-6	ATTGTATGAACAGCGATGATGCAC	CCAGGTAGAAACGGAACTCCAG
IL-1β	CCCTGAACTCAACTGTGAAATAGCA	CCCAAGTCAAGGGCTTGGAA
GAPDH	GAACATCATCCCTGCATCCA	CCAGTGAGCTTCCCGTTCA

### Western blot analysis

Total protein was extracted from injured spinal cord tissue stored at − 80 ℃ by using radioimmunoprecipitation assay (RIPA) lysis and extraction buffer that included a protease inhibitor cocktail. The concentration of protein was determined by the BCA method. Equal amounts of protein (30–50 µg) from each sample were separated using 4–20% SurePAGE™ Gels (Genscript) and transferred to nitrocellulose (NC) membranes (EMD Millipore Corp). The membranes were blocked with 5% bovine serum albumin for 1 h at room temperature and then incubated overnight at 4 °C with the following specific primary antibodies: rabbit anti-iNOS (18,985–1-AP, Proteintech, 1:1000), rabbit anti-C3 (ab200999, Abcam, 1:1000), rabbit anti-GFAP (SAB4300647, Sigma, 1:1000), rabbit anti-JAK2 (3230, Cell Signaling Technology, 1:1000), rabbit anti-pJAK2 (AF3022, Affinity, 1:1000), rabbit anti-STAT3 (12,640, Cell Signaling Technology, 1:1000), rabbit anti-pSTAT3 (ab76315, Abcam, 1:1000), rabbit anti-Lcn2 (ab63929, Abcam, 1:1000), and anti-β-actin (66,009–1-Ig, Proteintech, 1:1000). After incubation with the corresponding secondary antibodies (1:2000) for 1 h at room temperature, the membranes were scanned with ECL-Plus Reagent (Millipore) and observed under an Amersham Imager 600 (General Electric). The band intensity was analyzed by using ImageJ software (NIH).

### ELISA

The levels of C1q, IL-1α, and TNF-α in injured spinal cord tissue stored at − 80 ℃ at 3 dpi were evaluated with ELISA kits (Meimian Industrial Co., Ltd.). The injured spinal segments were homogenized in PBS, and the tissue homogenate supernatants were collected and analyzed according to the manufacturer's instructions.

### RNA‐seq analysis

RNA-seq experiments were performed to compare the sham control group and the SCI group at 1 dpi (*n* = 3 rats per group). The total RNA for each sample was separated and purified with TRIzol (Invitrogen) according to the operating protocol provided by the manufacturer. Then, a NanoDrop ND-1000 was used to determine the amount and purity of the total RNA. The integrity of the RNA was tested with an Agilent 2100 with an RIN > 7.0 as the qualifying standard, and agarose gel electrophoresis was used for verification. Five micrograms of total RNA was removed, and a Ribo-Zero™ rRNA Removal Kit (Illumina) was used to capture and remove ribosomal RNA according to standard operating procedures. Then, the linear RNA was removed by the action of RNase R (Epicentre Inc.), and the remaining RNA was fragmented with divalent cations at high temperature. Reverse transcriptase was used to synthesize cDNA from the fragmented RNA. Then, *E. coli* DNA polymerase I and RNase H were used for two-strand synthesis to convert these double-stranded DNA and RNA complexes into double-stranded DNA. At the same time, dUTPs were incorporated into the two strands to blunt the ends of the double-stranded DNA. Then, an A base was added at each end so that the DNA could be connected to a linker with a T base at the end, and magnetic beads were used to screen and purify the DNA fragments by size. The two strands were digested with UDG, and then PCR was used to form a library with a fragment size of 300 bp (± 50 bp). Finally, we used an Illumina HiSeq 4000 (LC Bio) to perform paired-end sequencing according to the standard operating procedures.

### Cell culture

Primary microglia and astrocytes were generated from postnatal (P0–P2) rat pups according to methods described previously with minor modifications [36]. Briefly, the cerebral cortex was carefully removed, cleaned of the meninges and the choroid plexus, dissected into 0.5-mm-thick slices, placed in 0.125% trypsin EDTA solution (Thermo Fisher Science) and shaken gently for 20 min in a 37 °C incubator. The digested tissue was centrifuged at 1000 rpm for 5 min and resuspended in DMEM/F12 (Gibco) with 10% fetal bovine serum (Gibco), 100 U/mL penicillin and 100 μg/mL streptomycin (Thermo Fisher Science). After filtration with 100 µm nylon mesh, the final single-cell suspension was cultured in a T75 culture flask that had been precoated with poly-L-lysine (Sigma Aldrich). The culture medium was exchanged the next day and then refreshed every three days. After culturing in vitro for 12–14 days, the culture flasks were shaken at 200 rpm for 2 h, and then mature microglia were obtained from the supernatant. The remaining astrocytes were digested with trypsin and recultured for follow-up experiments. Ventral spinal cord 4.1 (VSC4.1) motoneurons were cultured in DMEM (Gibco) supplemented with 10% fetal bovine serum (Gibco). All cells were incubated at 37 °C in a humidified incubator under 5% CO2.

### Cell treatment

Microglia with a neurotoxic phenotype were induced with lipopolysaccharide (LPS, 1 μg/ml, Sigma Aldrich) and interferon gamma (IFN-γ, 20 ng/ml, Sigma Aldrich). Astrocytes with a neurotoxic phenotype were induced with C1q (400 ng/ml, MyBioSource, MBS 143,105), TNF-α (30 ng/ml, Cell Signaling Technology, 8902SF) and IL-1α (3 ng/ml, Sigma, i3901). To detect the effect of PBM on induced microglia and astrocytes, the cells were treated with PBM (810 nm, 6 mW, 4.5 cm^2^, 8 min) immediately following induction and irradiated twice each day (every 12 h at fixed times of 9 am and 9 pm). To detect the neurotoxicity of induced microglia and astrocytes, microglia and astrocytes were induced for 24 h, and then the supernatants from the culture media were collected as microglia-conditioned medium (MCM) and astrocyte-conditioned medium (ACM), respectively. Then, half of the VSC4.1 cell culture medium was replaced with MCM or ACM, and the cells were cultured for another 24 h before detection of the apoptotic rates. After that, to explore the mechanism of activation of macroglia and astrocytes, we designed several experiments. In the first experiment, microglia and astrocytes were separately pretreated with cucurbitacin I (0.5 μM, MedChemExpress) for 1 h to inhibit the JAK2-STAT3 pathway. The cells were then induced for 24 h and treated or not treated with PBM. In the second experiment, an adenovirus carrying shRNA-LCN2 (HanBio) was used to knock down the levels of Lcn2 in microglia and astrocytes independently. Transfection was performed at a multiplicity of infection (MOI) of 20 for 24 h according to the manufacturer's instructions. To upregulate the level of Lcn2 in culture medium, recombinant Lcn2 protein (ReLcn2, 1 μg/ml, R&D Systems) was added to the medium of microglia and astrocytes separately at the same time as cell induction. Equal volumes of DMSO (without cucurbitacin I), adenoviral vector (without the target shRNA) and PBS (without ReLcn2) were used as controls. After induction for 24 h, cells were collected for subsequent experiments.

### Flow cytometry

To measure apoptosis, VSC4.1 cells treated with MCM or ACM for 24 h were collected for dual staining with propidium iodide (PI) and Alexa Fluor 488-annexin V using an Alexa Fluor 488-annexin V Dead Cell Apoptosis Kit (Thermo Fisher) according to the manufacturer’s protocol. The stained cells were analyzed by flow cytometry (Beckman F500), and at least 10,000 cells in each sample were analyzed.

### Statistical analysis

All data are presented as the means ± standard deviations (means ± SDs). Statistical analysis was performed with GraphPad Prism 8 software. One-way analysis of variance (ANOVA) followed by Bonferroni’s post hoc test was used to test differences between groups at specific times. Two-way repeated measures ANOVA followed by Bonferroni’s post hoc test was used to test differences between groups at different times. Statistical significance was defined at *p* < 0.05.

## Results

### 1. PBM promoted motor function recovery, reduced apoptosis and increased the number of surviving neurons after SCI

**Fig. 1:**
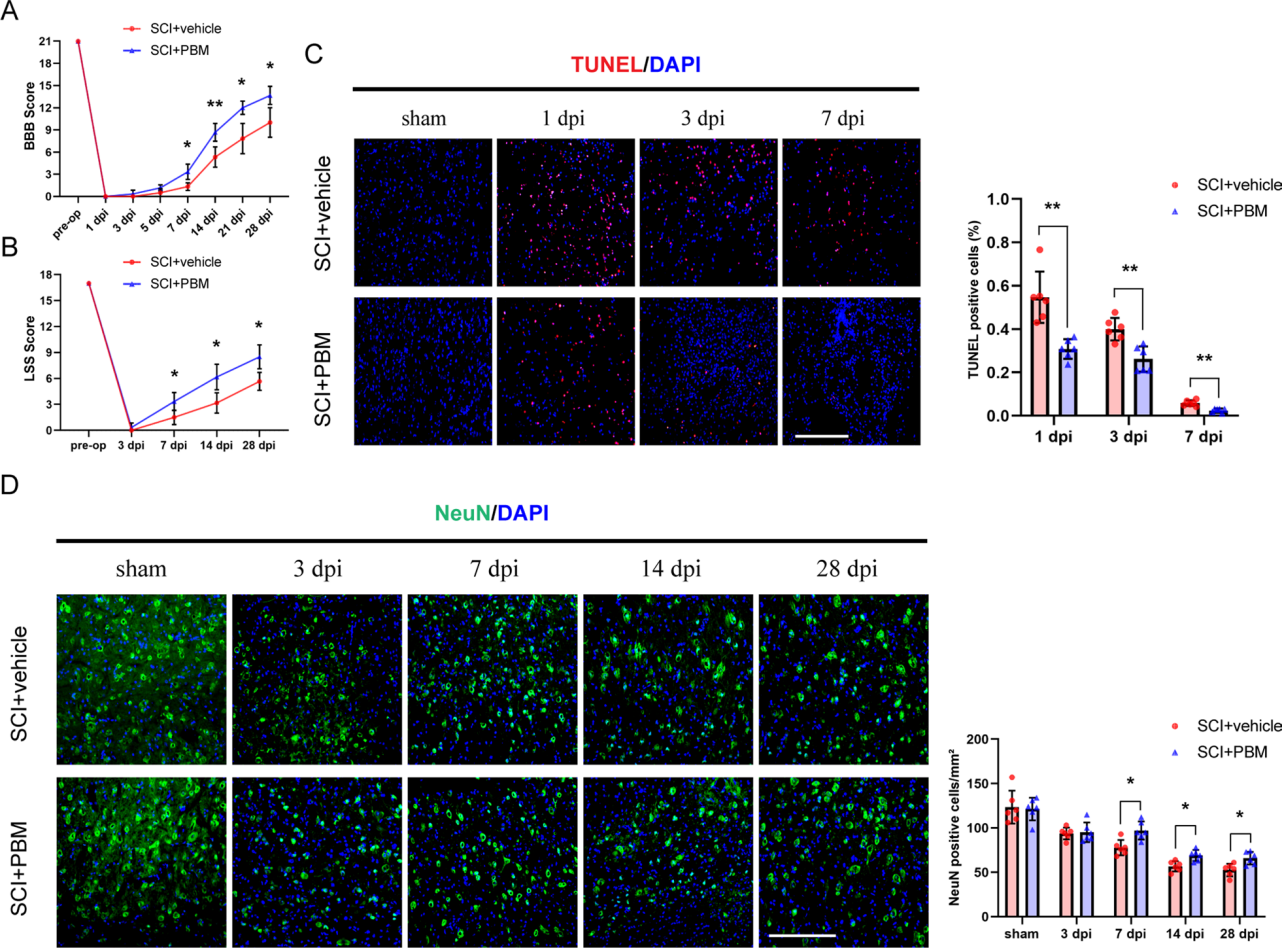
PBM promoted the recovery of motor function, reduced tissue apoptosis, and increased the number of surviving neurons after SCI. **A, B** The BBB score and LSS were used to evaluate the recovery of motor function in the SCI + vehicle group and the SCI + PBM group (*n* = 6 rats per group). **C** TUNEL staining was used to detect the level of apoptosis in injured spinal cord tissue at 1 dpi, 3 dpi and 7 dpi. dpi: days post-injury. Representative images were obtained within ± 150 μm of the lesion epicenter in the ventral horn of the spinal cord. The apoptotic rate is represented by the number of TUNEL^+^ cells divided by the total number of cells in which nuclei were labeled in each field. Quantification of the TUNEL^+^ cell rate in the SCI + vehicle group and the SCI + PBM group (*n* = 6 rats per group at each time point). **D** The number of surviving neurons in the ventral spinal cord gradually decreased within 28 dpi. Representative images were obtained within ± 150 μm of the lesion epicenter in the ventral horn of the spinal cord. Quantification of the number of NeuN^+^ cells in the SCI + vehicle group and the SCI + PBM group (*n* = 6 rats per group at each time point). Statistical comparisons were performed using two-way ANOVA followed by Bonferroni’s post hoc test. *p < 0.05, **p < 0.01

The BBB scale and the LSS were used to comprehensively evaluate the effect of PBM on the recovery of motor function after SCI. The BBB score results showed that the difference between the SCI + PBM group and the SCI + vehicle group began to appear at 7 dpi (Fig. 1A, F (7, 70) = 12.69, p < 0.0001. SCI + PBM group vs. SCI + vehicle group, 3.33 ± 1.03 vs. 1.33 ± 0.52, *p* = 0.0274, 7 dpi). The LSS showed similar results: the score in the SCI + PBM group was higher than that in the SCI + vehicle group at 7 dpi (Fig. 1B; F (4, 40) = 10.83, p < 0.0001. SCI + PBM group vs. SCI + vehicle group, 3.33 ± 1.03 vs. 1.50 ± 0.84, *p* = 0.0372, 7 dpi).

Next, we investigated tissue apoptosis in each group and found that apoptosis mainly occurred in the acute phase (within 7 dpi). No TUNEL-positive cells were detected in the sham control group. As shown in Fig. 1C, the proportions of TUNEL-positive cells in the SCI + PBM group vs. the SCI + vehicle group (F (2, 20) = 10.12, *p* = 0.0009) were 30.8% ± 4.6% vs. 54.7% ± 11.8% at 1 dpi (*p* = 0.0090), 26.2% ± 5.8% vs. 40.0% ± 5.1% at 3 dpi (*p* = 0.0047), and 2.5% ± 0.8% vs. 5.9% ± 1.3% at 7 dpi (*p* = 0.0018). We also counted surviving motor neurons in the ventral spinal cord, which are essential to motor function. The results showed that there were more neurons in the SCI + PBM group than in the SCI + vehicle group at 7 dpi (Fig. 1D, F (4, 40) = 2.177, *p* = 0.0890. SCI + PBM group vs. SCI + vehicle group, 97.17 ± 10.19 vs. 77.83 ± 8.59, *p* = 0.0273). Similarly, at 14 dpi and 28 dpi, the ability to retain motor neurons after injury was significantly different between the SCI + PBM group and the SCI + vehicle group (69.00 ± 6.23 vs. 56.83 ± 5.78, *p* = 0.0285, 14 dpi; 66.00 ± 7.15 vs. 52.67 ± 7.12, *p* = 0.0447, 28 dpi).

### 2.Effects of PBM on the dynamic changes in microglia/macrophages and astrocytes after SCI

**Fig. 2:**
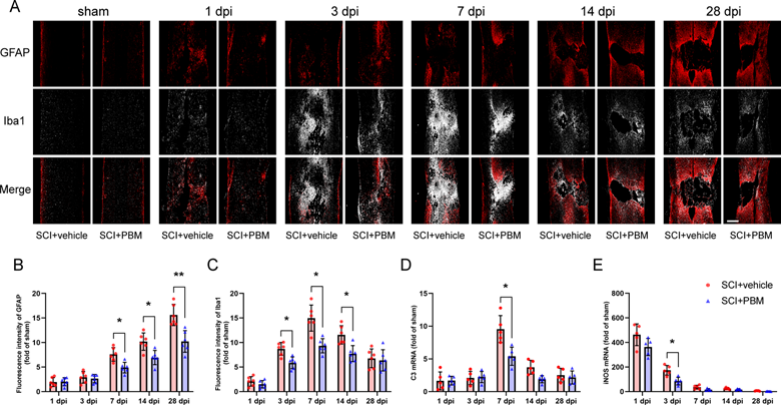
Effects of PBM on the dynamic changes in microglia/macrophages and astrocytes after SCI. **A** Representative images of immunofluorescence staining for GFAP (red) and Iba1 (white) in injured spinal cord sections in the SCI + vehicle group and the SCI + PBM group (at 1 dpi, 3 dpi, 7 dpi, 14 dpi and 28 dpi). GFAP: glial fibrillary acidic protein; Iba1: ionized calcium binding adapter molecule 1. **B, C** Quantification of the mean fluorescence intensity of GFAP^+^ and Iba1^+^ cells (*n* = 6 rats per group at each time point). **D, E** Relative mRNA expression of C3 and iNOS presented as the fold change in comparison to the level in the sham control group (*n* = 5 rats per group). C3: complement C3; iNOS, inducible nitric oxide synthase. Scale bar: 400 μm. Statistical comparisons were performed using two-way ANOVA followed by Bonferroni’s post hoc test. *p < 0.05, **p < 0.01

Subsequently, we examined the distribution and activation of microglia/macrophages (Iba1^+^ cells) and astrocytes (GFAP^+^ cells) in spinal cord tissue on different days after injury. As shown in Fig. 2a, the normal and complete tissue structure was destroyed, and microglia/macrophages began to activate and proliferate within 24 h after SCI. By 3 dpi, a large number of microglia/macrophages had been recruited to the damaged area. The number of microglia peaked at 7 dpi. When inflammation entered the subacute phase, astrocytes started to take over microglia/macrophages, significantly upregulating the expression of GFAP, and the two types of glial cells jointly participated in scar formation. By 28 dpi, the spinal cord cavity had formed, and the scar structure had become denser. Compared with vehicle treatment, PBM downregulated the expression levels of Iba1 and GFAP post SCI (Fig. 2B, F (4, 40) = 7.217, *p* = 0.0002. *p* = 0.0201, 7 dpi; *p* = 0.0312, 14 dpi; *p* = 0.0081, 28 dpi. Figure 2C, F (4, 40) = 4.796, *p* = 0.0030. *p* = 0.0145, 3 dpi; *p* = 0.0100, 7 dpi; *p* = 0.0191, 14 dpi).

To further explore the activation of neurotoxic microglia/macrophages and astrocytes, we investigated the mRNA expression of C3 and iNOS by RT-PCR. The results suggested that the expression of C3 began to peak at 7 dpi, while PBM suppressed this upregulation trend (Fig. 2D, F (4, 32) = 7.605, *p* = 0.0002. *p* = 0.0368, 7 dpi). The level of iNOS immediately increased at 1 dpi and then gradually decreased, and PBM significantly inhibited the upregulation of iNOS at 3 dpi (Fig. 2E, F (4, 32) = 3.112, *p* = 0.0285. *p* = 0.0256, 3 dpi).

### 3. PBM inhibited the activation of neurotoxic microglia/macrophages after SCI

**Fig. 3:**
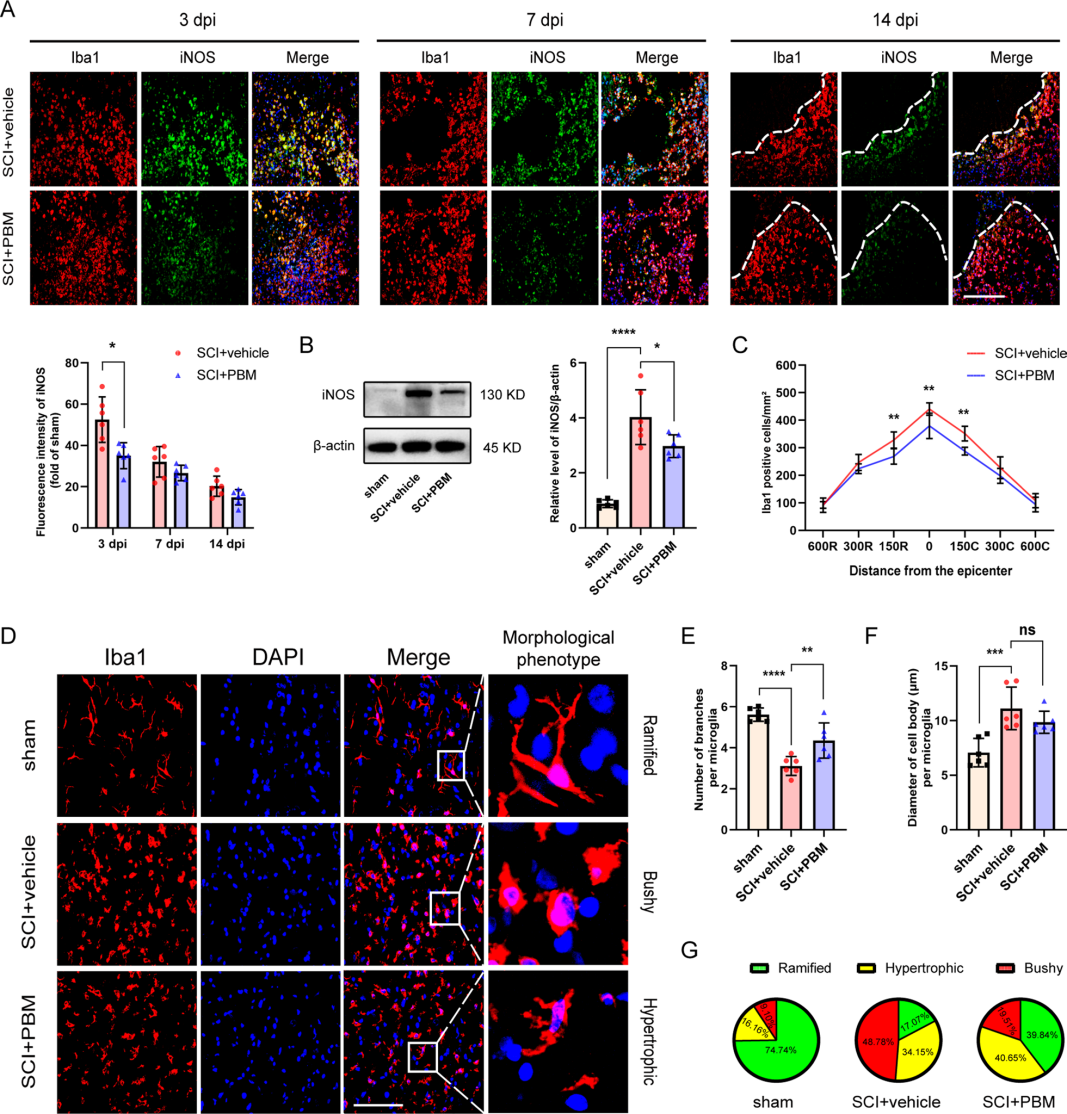
PBM inhibited the activation of neurotoxic microglia/macrophages after SCI. **A** Representative images of immunofluorescence staining for Iba1 (red) and iNOS (green) in from the lesion epicenter at 3 dpi, 7 dpi, and 14 dpi. Quantification of the intensity of iNOS relative to that in the sham control group (*n* = 6 rats per group at each time point). Statistical comparisons were performed using two-way ANOVA followed by Bonferroni’s post hoc test. **B** Representative blots and quantification showing the expression level of iNOS in the injured spinal cord at 3 dpi (*n* = 6 rats per group). Statistical comparisons were performed using one-way ANOVA followed by Bonferroni’s post hoc test. **C** Quantification of Iba1^+^ cells at different distances from the epicenter from rostral to caudal (600 μm, 300 μm, and 150 μm; *n* = 6 rats per group). Statistical comparisons were performed using two-way ANOVA followed by Bonferroni’s post hoc test. **D** Representative images of Iba1^+^ cells at 150 μm from the epicenter in each group at 3 dpi. Microglia/macrophages were classified into ramified, bushy and hypertrophic phenotypes based on cellular morphological features. **E, F** Iba1^+^ cell morphology was assessed by the number of branches per cell and the diameter of the cell body per cell (*n* = 6 rats per group). Statistical comparisons were performed using one-way ANOVA followed by Bonferroni’s post hoc test. **G** Representative percentages of ramified, hypertrophic and bushy microglial phenotypes in each group at 3 dpi. Scale bar: 200 μm. *p < 0.05, **p < 0.01, ***p < 0.001, ****p < 0.0001, ns = nonsignificant

Considering that microglial/macrophage activation was most obvious in the acute phase after injury, we first observed changes in microglia/macrophages (Fig. 3A). At 3 dpi, a large number of microglia/macrophages had high iNOS expression (neurotoxic type) in the injury epicenter in the SCI + vehicle group, while the intensity of iNOS in the SCI + PBM group was weaker (F (2, 20) = 4.609, *p* = 0.0226; *p* = 0.0293, 3 dpi). Western blot assays showed the same results (Fig. 3B, F (2, 15) = 39.06, *p* < 0.0001. SCI + PBM group vs. SCI + vehicle group, *p* = 0.0325). At 7 dpi and 14 dpi, we also observed that the intensity of iNOS was decreased in the SCI + PBM and SCI + vehicle groups, but there was no significant difference between the two groups (Fig. 3A, *p* = 0.4569, 14 dpi; *p* = 0.1809, 28 dpi).

There were differences in the spatial distribution of microglia/macrophages after SCI. Therefore, we separately counted the Iba1^+^ cells at distances of 150 μm, 300 μm, and 600 μm from the epicenter to the rostral or caudal sides at 3 dpi. The results showed that PBM reduced the number of Iba1^+^ cells within 150 μm of the epicenter in the rostral and caudal directions in the spinal cord (Fig. 3C, F (6, 70) = 2.601, *p* = 0.0247. SCI + PBM group vs. SCI + vehicle group, 268.56 ± 28.05 vs. 328.26 ± 29.61, *p* = 0.0028, at 150 μm rostral direction; 379.95 ± 46.20 vs. 440.71 ± 22.30, *p* = 0.0023, at epicenter; 287.66 ± 13.88 vs. 351.51 ± 26.91, *p* = 0.0012, at 150 μm caudal direction).

We next investigated the morphology of microglia/macrophages within 150 μm of the center of the injury area (Fig. 3D). Ramified microglia/macrophages are in a homeostatic state, while bushy and hypertrophic microglia/macrophages represent a typical activated phenotype. In the sham control group, ramifications with reduced cell diameters and increased numbers of bifurcations were dominant. After injury, most of the cell branches were reduced in number, the cell bodies were hypertrophic, and the proportions of bushy and hypertrophic microglial cells were increased. PBM inhibited the activation of microglia/macrophages, as indicated by evaluation of the numbers of branches and the diameters of the cell bodies of microglia (Fig. 3E, F (2, 15) = 26.73, *p* < 0.0001. SCI + PBM group vs. SCI + vehicle group, *p* = 0.0076; Fig. 3F, F (2, 15) = 11.96, *p* = 0.0008. SCI + PBM group vs. SCI + vehicle group, *p* = 0.4703). PBM reduced the total ratio of bushy- and hypertrophic-phenotype cells after injury (Fig. 3G).

### 4. PBM inhibited the activation of neurotoxic astrocytes after SCI

**Fig. 4:**
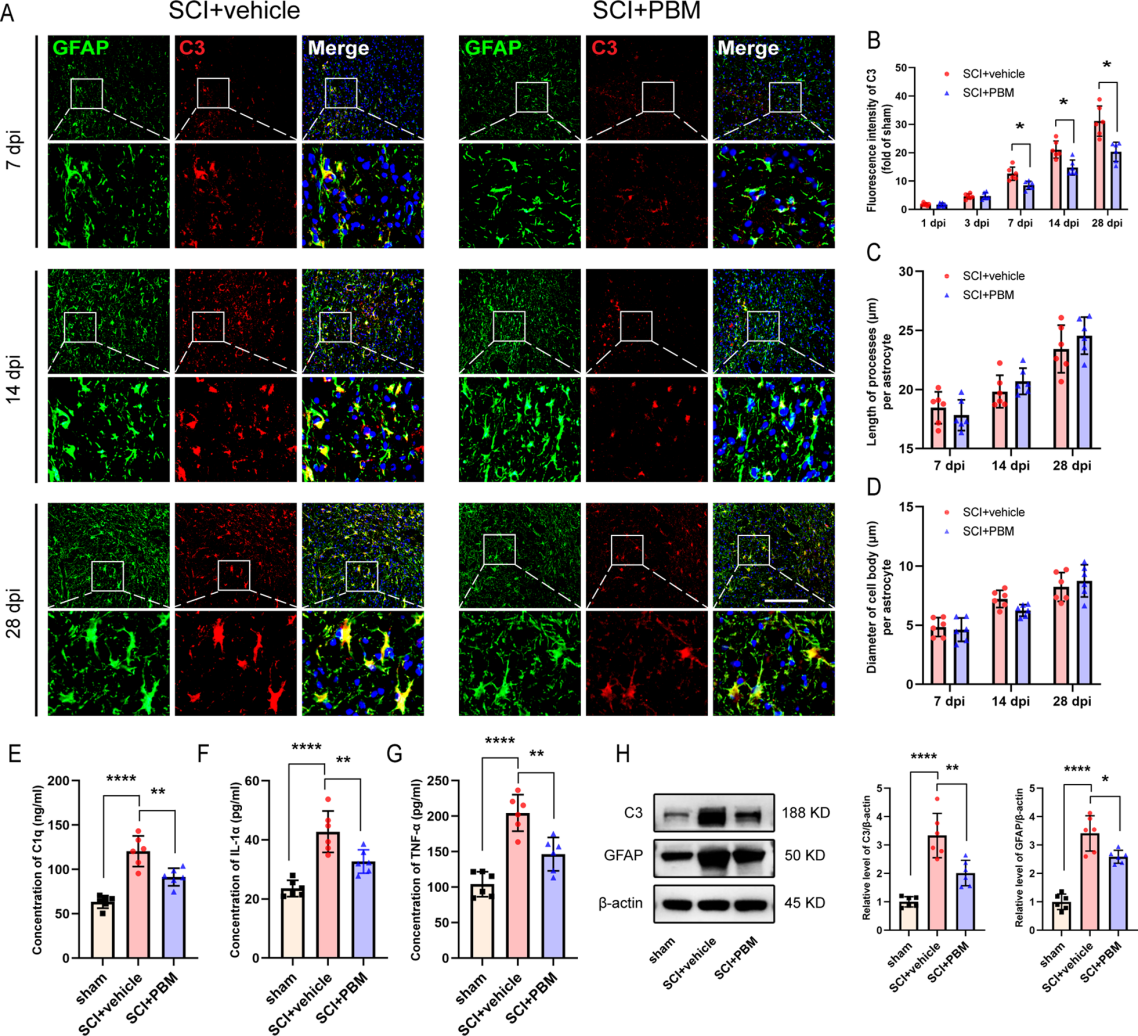
PBM inhibited the activation of neurotoxic astrocytes after SCI. **A** Representative images of immunofluorescence staining for GFAP (green) and C3 (red) taken from an area 150 μm from the epicenter in the SCI + vehicle and SCI + PBM groups at 7 dpi, 14 dpi and 28 dpi. **B** Quantification of the fluorescence intensity of C3 in the SCI + vehicle group and the SCI + PBM group relative to the sham control group (*n* = 6 rats per group at each time point). Statistical comparisons were performed using two-way ANOVA followed by Bonferroni’s post hoc test. **C**, **D** Astrocytic morphology was assessed in terms of the length of processes per cell and the diameter of the cell body per astrocyte (*n* = 6 rats per group at each time point). Statistical comparisons were performed using two-way ANOVA followed by Bonferroni’s post hoc test. **E**–**G** ELISA results for C1q, IL-1α and TNF-α in the injured spinal cords of each group at 3 dpi (*n* = 6 rats per group). Statistical comparisons were performed using one-way ANOVA followed by Bonferroni’s post hoc test. **H** Representative blots and quantification showing the expression levels of C3 and GFAP in each group at 7 dpi (*n* = 6 rats per group). Statistical comparisons were performed using one-way ANOVA followed by Bonferroni’s post hoc test. Scale bar: 200 μm. *p < 0.05, **p < 0.01, ****p < 0.0001

Neurotoxic astrocytes are induced by C1q, IL-1α, and TNF-α secreted by activated microglia [6]. We investigated the protein expression levels in the injured spinal cord tissue at 3 dpi by ELISA and found that the levels of C1q, IL-1α and TNF-α were significantly higher in the injury group than in the sham control group. PBM inhibited the increasing trend (Fig. 4E, F (2, 15) = 32.82, *p* < 0.0001. SCI + PBM group vs. SCI + vehicle group, 91.50 ± 9.97 vs. 120.50 ± 17.19 ng/ml, *p* = 0.0027; Fig. 4F, F (2, 15) = 23.00, *p* < 0.0001. SCI + PBM group vs. SCI + vehicle group, 32.69 ± 3.97 vs. 42.77 ± 6.98 pg/ml, *p* = 0.0085; Fig. 4G, F (2, 15) = 29.90, *p* < 0.0001. SCI + PBM group vs. SCI + vehicle group, 146.50 ± 23.52 vs. 204.50 ± 25.78 pg/ml, *p* = 0.0014).

By 7 dpi, neurotoxic astrocytes were obviously activated, and a large number of C3^+^ cells had appeared in the injured area (Fig. 4A). As the injury course entered the subacute phase, the expression of C3 was also upregulated and basically colocalized only with GFAP^+^ cells. After PBM, the expression of GFAP and C3 (Fig. 4B) was effectively inhibited (F (4, 40) = 8.491, *p* < 0.0001. *p* = 0.0309, 7 dpi; *p* = 0.0164, 14 dpi; *p* = 0.01287, 28 dpi). Western blot assays also showed that the C3 and GFAP protein levels in the SCI + PBM group were lower than those in the SCI + vehicle group at 7 dpi (Fig. 4H, C3: F (2, 15) = 29.26, *p* < 0.0001. SCI + PBM group vs. SCI + vehicle group, *p* = 0.0019; GFAP: F (2, 15) = 52.81, *p* < 0.0001. SCI + PBM group vs. SCI + vehicle group, *p* = 0.0106). However, when we measured the process lengths and the cell body diameters of astrocytes, there were no significant differences between the SCI + vehicle group and the SCI + PBM group at 3 dpi, 7 dpi, or 14 dpi (Fig. 4C, F (2, 20) = 1.423, *p* = 0.2644; Fig. 4D, F (2, 20) = 2.208, *p* = 0.1360).

### 5. Lcn2 upregulation and JAK2-STAT3 pathway activation were suppressed by PBM after SCI

**Fig. 5:**
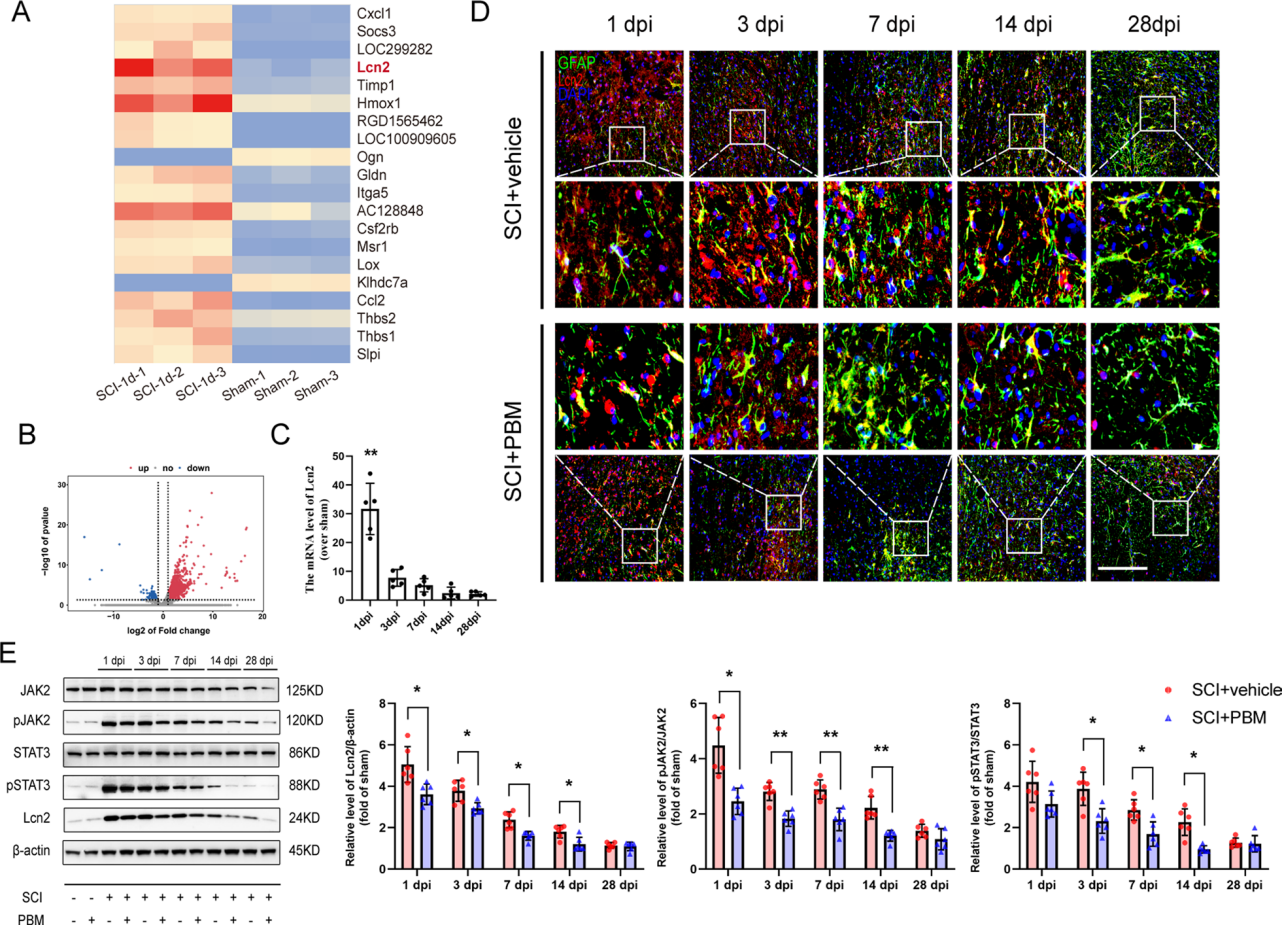
PBM suppressed the upregulation of Lcn2 and the activation of the JAK2-STAT3 pathway after SCI. **A**, **B** Upregulated (1379) and downregulated (168) genes in the sham group and the SCI group at 1 dpi. The heatmap indicates that Lcn2 was one of the highly upregulated genes. **C** The relative mRNA expression of Lcn2 is presented as the fold change in each group after SCI in comparison to the expression in the sham control group (*n* = 5 rats per group). Statistical comparisons were performed using one-way ANOVA followed by Bonferroni’s post hoc test. **D** Representative images of immunofluorescence staining for GFAP (green), Lcn2 (red) and DAPI (blue) taken from the lesion area 150 μm from the epicenter in the SCI + vehicle group and the SCI + PBM group at 1 dpi, 3 dpi, 7 dpi, 14 dpi and 28 dpi. e. Representative blots showing the expression levels of JAK2, pJAK2, STAT3, pSTAT3 and Lcn2 in each group at 1 dpi, 3 dpi, 7 dpi, 14 dpi and 28 dpi. Quantification of the relative expression of Lcn2, pJAK2/JAK2 and pSTAT3/STAT3 (*n* = 6 rats per group at each time point). Statistical comparisons were performed using two-way ANOVA followed by Bonferroni’s post hoc test. Scale bar: 200 μm. *p < 0.05, **p < 0.01

Within 24 h after SCI, large amounts of proinflammatory factors were released from the injured area, causing an inflammatory storms. RNA-seq analysis showed that 1,379 genes were upregulated and 168 genes were downregulated in the SCI group and sham groups at 1 dpi. Lcn2 was one of the significantly upregulated genes (Fig. 5A, B). Lcn2 is a member of the secreted lipocalin protein family that is upregulated in CNS diseases or injury contexts and aggravates neuroinflammation [37]. Lcn2 is synthesized and secreted as an inducible factor from activated microglia and reactive astrocytes, and it has been recognized as a modulatory factor for diverse cellular processes, such as cell death, survival, morphology, migration, invasion, differentiation, and functional polarization [21, 38]. We next assessed the expression of Lcn2 in the tissues on different days after injury. The RT-PCR results showed that the Lcn2 expression in the SCI group was dramatically increased at 1 dpi (Fig. 5C, F (5, 24) = 41.76, *p* < 0.0001), which was consistent with the results of immunofluorescence staining. Lcn2 expression decreased gradually within 28 dpi. On different days after injury, the fluorescence intensity of Lcn2 in the SCI + PBM group was lower than that in the SCI + vehicle group (Fig. 5D).

Considering the important role of JAK2-STAT3 signaling pathway activation in Lcn2 regulation and glial cell activation after SCI, we next examined the activation of the JAK2-STAT3 pathway in the SCI + PBM and SCI + vehicle groups. Western blot analysis showed that the activation time course of the JAK2-STAT3 pathway after SCI paralleled the course of Lcn2 expression. In contrast, PBM inhibited the activation of the JAK2-STAT3 pathway, which was consistent with the suppression of Lcn2 (Fig. 5E, Lcn2: F (4, 40) = 3.996, *p* = 0.0081; pJAK2/JAK2: F (4, 40) = 6.051, *p* = 0.0007; pSTAT3/STAT3: F (4, 40) = 3.854, *p* = 0.0097).

### 6. PBM inhibited the activation of microglia and astrocytes in vitro

**Fig. 6:**
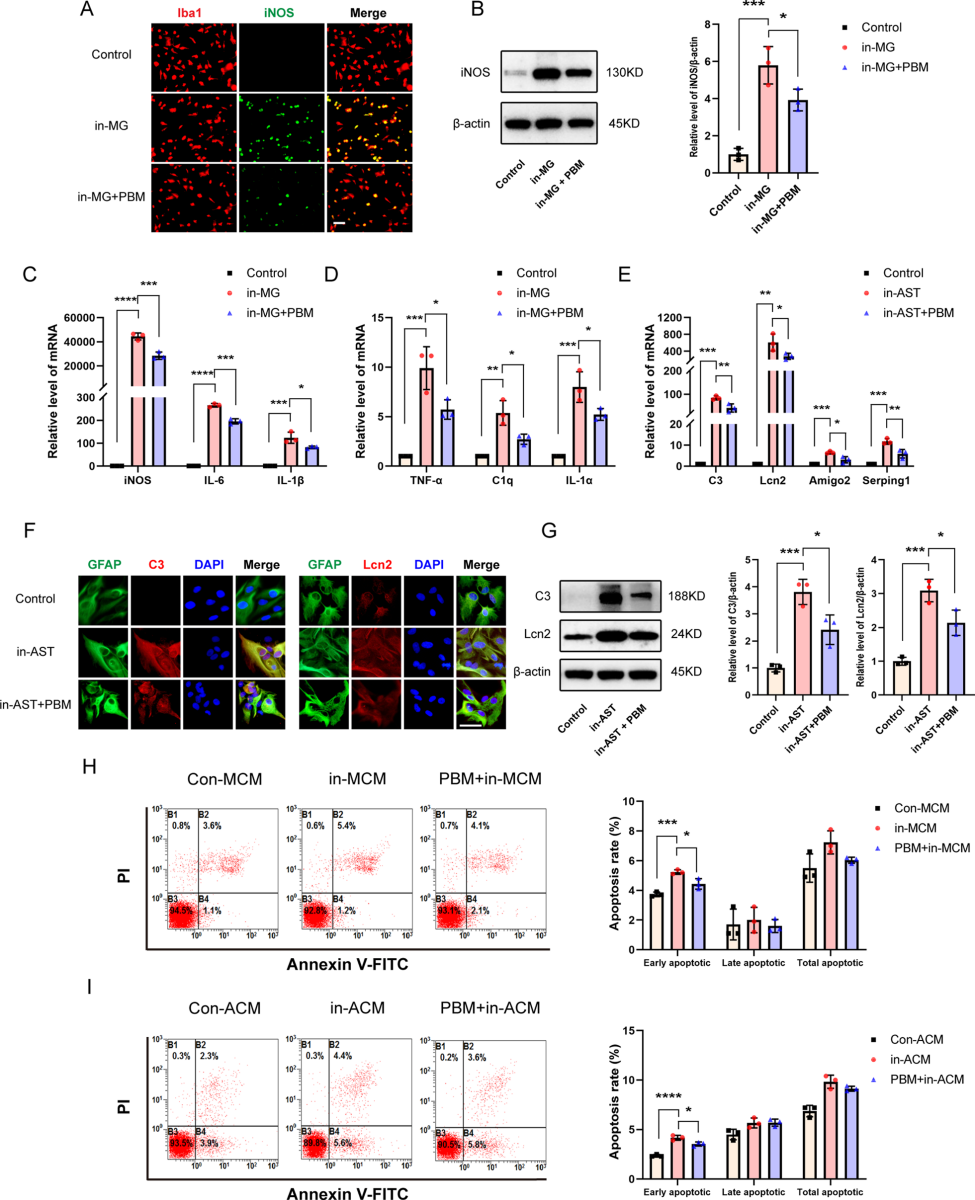
PBM inhibited the activation of microglia and astrocytes in vitro. **A** Representative images of immunofluorescence staining for Iba1 (red) and iNOS (green) in the control group, the in-MG group and the in-MG + PBM group. In-MG: induced microglia. **B** Western blotting was used to detect the protein levels of iNOS in each group. Quantification of the relative expression of iNOS in comparison to the expression in the control group. **C**, **D** RT-PCR was used to detect the mRNA levels of iNOS, IL-6, IL-1β, TNF-α, C1q and IL-1α in each group. **e** RT-PCR was used to detect the mRNA levels of C3, Lcn2, Amigo2 and Serping1 in the control group, the in-AST group and the in-AST group. In-AST: induced astrocyte. **F** Representative images of immunofluorescence staining for GFAP (green) and C3 (red) or Lcn2 (red) in the control group, the in-AST group and the in-AST group. **G** Western blotting was used to detect the protein levels of C3 and Lcn2 in each group. Quantification of the relative expression of C3 and Lcn2 in comparison to the expression in the control group. **H, I** Apoptosis was measured by flow cytometry. B1, B2, B3, and B4 represent quadrants for dead, late apoptotic, viable, and early apoptotic cells, respectively. Early apoptosis, not late apoptosis or total apoptosis, was induced in VSC4.1 cells in the in-MCM group and in-AST group, but this effect was alleviated by two pretreatments with PBM. Con-MCM: control microglia-conditioned medium; Con-ACM: control astrocyte-conditioned medium; in-MCM: induced microglia-conditioned medium; in-ACM: induced astrocyte-conditioned medium. Scale bar: 200 mm. The experiments were independently repeated three times. Statistical comparisons were performed using one-way ANOVA followed by Bonferroni’s post hoc test. *p < 0.05, **p < 0.01, ***p < 0.001, ****p < 0.0001

To better verify the mechanism of PBM, we simulated neuroinflammatory conditions in vitro to activate microglia and astrocytes. We cultured primary microglia and astrocytes; used LPS and IFN-γ to induce microglial activation; and used C1q, TNF-α and IL-1α to induce astrocyte activation. Then, laser irradiation was applied with optimized parameters in order to verify the effect of PBM on neurotoxic microglia and astrocytes. The irradiation had no obvious effects on resting cells, as previously shown by our team (data not shown). The immunofluorescence and Western blot results showed that the expression of iNOS in induced microglia (in-MG) was significantly upregulated, while PBM inhibited this upregulation (Fig. 6A, B, F (2, 6) = 36.29, *p* = 0.0004. In-MG + PBM group vs. in-MG group, *p* = 0.0494). RT-PCR showed that proinflammatory factors such as iNOS, IL-6, and IL-1β, as well as C1q, IL-1α, and TNF-α, were all upregulated after induction. PBM inhibited the upregulation of inflammatory indicators to varying degrees (Fig. 6C, iNOS: F (2, 6) = 259.9, *p* < 0.0001; IL-6, F (2, 6) = 885.3, *p* < 0.0001; IL-1β, F (2, 6) = 56.98, *p* = 0.0001. Figure 6D, C1q, F (2, 6) = 31.71, *p* = 0.0006; IL-1α, F (2, 6) = 24.27, *p* = 0.0013; TNF-α, F (2, 6) = 40.67, *p* = 0.0003).

Immunofluorescence staining and Western blot analysis showed that in addition to the expression of C3, which was high in induced astrocytes (in-AST) in vitro, the expression of Lcn2 increased significantly. After PBM, the increases in C3 and Lcn2 were inhibited (Fig. 6F, G, C3: F (2, 6) = 33.31, *p* = 0.0006. In-AST + PBM group vs. in-AST group, *p* = 0.0202; Lcn2, F (2, 6) = 37.13, *p* = 0.0004. In-AST + PBM group vs. in-AST group, *p* = 0.0236). We also assessed the reactive astrocyte-specific transcripts Amigo2 and Serping1. Similar to findings regarding the expression of C3 and Lcn2, PBM blocked the high mRNA expression of these molecules in induced astrocytes (Fig. 6E, C: F (2, 6) = 41.34, *p* = 0.0003; Lcn2, F (2, 6) = 18.91, *p* = 0.0026; Amigo2, F (2, 6) = 28.24, *p* = 0.0009; Serping1, F (2, 6) = 44.34, *p* = 0.0003).

Next, we used VSC4.1 motoneurons to verify the neurotoxic effects of induced microglia and astrocytes and to determine whether PBM could attenuate these neurotoxic effects. We added MCM and ACM to VSC4.1 cells separately and performed flow cytometry to detect the apoptosis levels in VSC4.1 cells (Fig. 6H, I). The results showed that when VSC4.1 cells were incubated with induced MCM (in-MCM) for 24 h, the early apoptotic rate was 5.23 ± 0.15%, the late apoptotic rate was 2.00 ± 0.85%, and the total apoptotic rate was 7.23 ± 0.78%. However, irradiation of the induced microglia twice daily (every 12 h) before the medium was collected alleviated the neurotoxic effects of MCM. Flow cytometry revealed that the early apoptotic rate was 4.43 ± 0.35% (vs. in-MCM group, F (2, 6) = 29.82, *p* = 0.0008), the late apoptotic rate was 1.60 ± 0.44% (vs. in-MCM group, F (2, 6) = 0.1950, *p* = 0.8278), and the total apoptotic rate was 6.03 ± 0.21% (vs. in-MCM group, F (2, 6) = 4.557, *p* = 0.0626). The effect of PBM on ACM was similar; the high rate of early apoptosis in VSC4.1 cells caused by induced ACM (in-ACM) was reversed after PBM. The early apoptotic rate in the PBM + in-ACM group was 3.53 ± 0.21% (vs. in-ACM group, 4.17 ± 0.25%, F (2, 6) = 62.53, *p* < 0.0001), the late apoptotic rate in the PBM + in-ACM group was 5.70 ± 0.36% (vs. in-ACM group, 5.67 ± 0.53%, F (2, 6) = 6.337, *p* = 0.0332), and the total apoptotic rate in the PBM + in-ACM group was 9.13 ± 0.25% (vs. in-ACM group, 9.83 ± 0.67%, F (2, 6) = 25.77, *p* = 0.0011).

### 7. PBM interfered with Lcn2/JAK2-STAT3 crosstalk during the activation of neurotoxic microglia and astrocytes in vitro

**Fig. 7:**
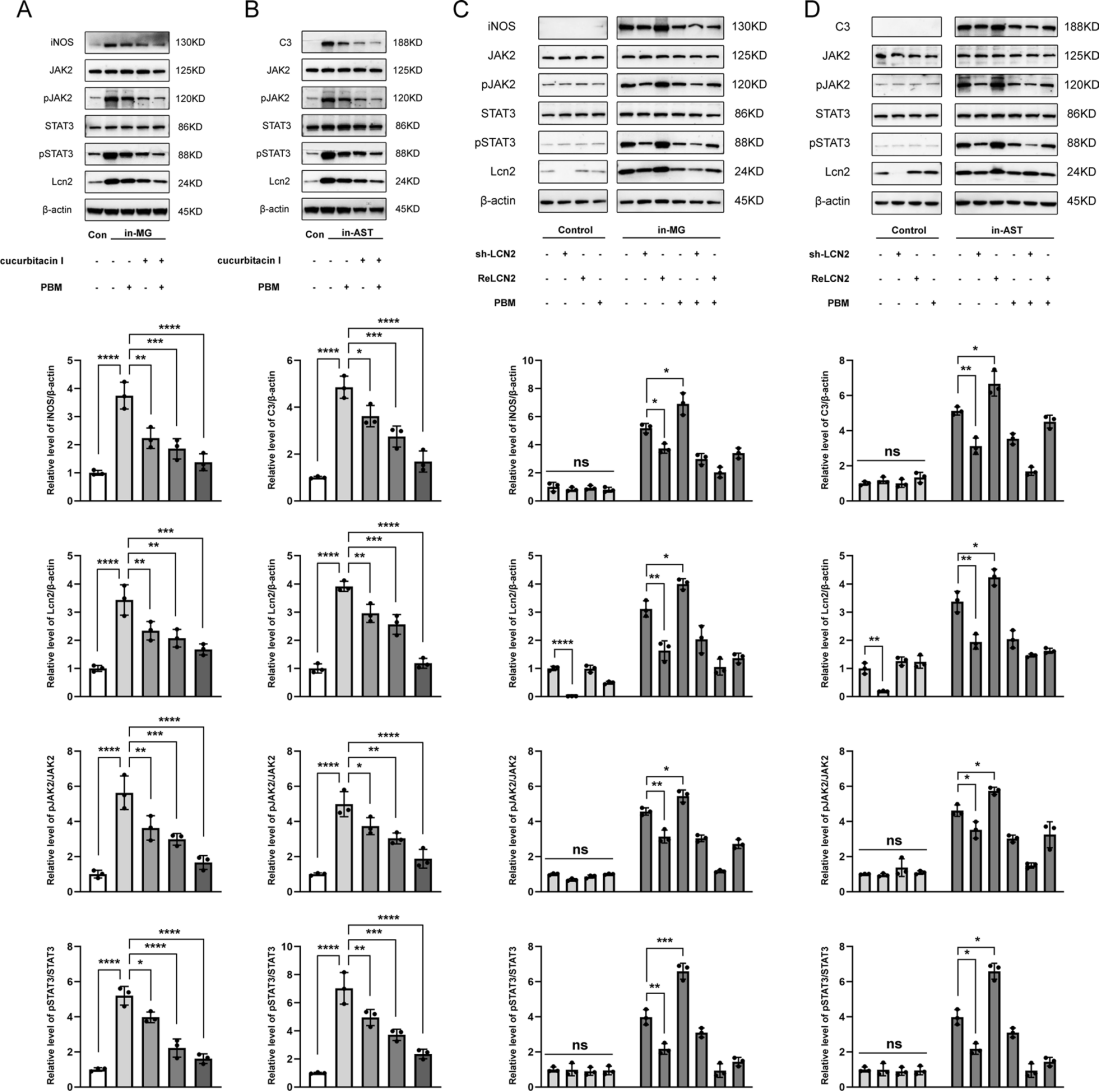
PBM interfered with Lcn2/JAK2-STAT3 crosstalk during the activation of neurotoxic glial cells in vitro. PBM and cucurbitacin I synergistically inhibited the activation of microglia (**A**) and astrocytes (**B**). Representative blots showing the expression levels of C3 (or iNOS), Lcn2, JAK2, pJAK2, STAT3 and pSTAT3 in each group. Quantification of the relative levels of C3 (or iNOS), Lcn2, pJAK2/JAK2, and pSTAT3/STAT3. Induced microglia (**C**) and induced astrocytes (**D**) were treated with PBM and transfected with an adenovirus to knock down Lcn2 or treated with ReLcn2, respectively. Representative blots showing the expression levels of C3 (or iNOS), Lcn2, JAK2, pJAK2, STAT3 and pSTAT3 in each group. Quantification of the relative levels of C3 (or iNOS), Lcn2, pJAK2/JAK2, and pSTAT3/STAT3. The experiments were independently repeated three times. Statistical comparisons were performed using one-way ANOVA followed by Bonferroni’s post hoc test. *p < 0.05, **p < 0.01, ***p < 0.001, ****p < 0.0001

The results of animal experiments suggested that the mechanism of PBM may be related to the inhibition of high Lcn2 expression and the activation of the JAK2-STAT3 signaling pathway after SCI. To explore the relationship between these two factors, we designed in vitro experiments. First, we used cucurbitacin I in vitro to specifically block the JAK2-STAT3 pathway before the induction of microglia and astrocytes. Similar to PBM, application of cucurbitacin I alone reduced the levels of C3 and iNOS while also attenuating the increase in Lcn2 with cell induction. Combined application of PBM and cucurbitacin I resulted in enhanced inhibition of microglial and astrocyte activation (Fig. 7A, iNOS: F (4, 10) = 28.74, *p* < 0.0001; Lcn2: F (4, 10) = 22.02, *p* < 0.0001; pJAK2/JAK2: F (4, 10) = 28.80, *p* < 0.0001; pSTAT3/STAT3: F (4, 10) = 62.39, *p* < 0.0001. Figure 7B, C3: F (4, 10) = 42.03, *p* < 0.0001; Lcn2: F (4, 10) = 71.25, *p* < 0.0001; pJAK2/JAK2: F (4, 10) = 32.32, *p* < 0.0001; pSTAT3/STAT3: F (4, 10) = 43.44, *p* < 0.0001).

Next, we altered Lcn2 levels in microglia and astrocytes via adenovirus transfection and recombinant protein application. The results showed that when microglia and astrocytes were not induced, the levels of Lcn2 and JAK2-STAT3 pathway activation were relatively low, and adenovirus transfection effectively reduced the level of Lcn2. After PBM and ReLcn2 were applied to resting microglia and astrocytes, respectively, there were no significant changes in the expression of Lcn2, the activation of JAK2-STAT3, or the level of iNOS (or C3). In induced microglia and astrocytes, knocking down Lcn2 alone inhibited the high expression of iNOS (or C3) and simultaneously blocked the activation of the JAK2-STAT3 pathway. The addition of ReLcn2 to the culture medium further increased the activation of the JAK2-STAT3 pathway and of neurotoxic microglia and astrocytes. Cotreatment with PBM enhanced the inhibitory effect of shRNA-Lcn2, while ReLcn2 weakened the effect of PBM (Fig. 7C, iNOS: F (2, 6) = 27.73, *p* = 0.0009; Lcn2: F (2, 6) = 53.13, *p* = 0.0002; pJAK2/JAK2: F (2, 6) = 40.33, *p* = 0.0003; pSTAT3/STAT3: F (2, 6) = 91.99, P < 0.0001. Figure 7D, C3: F (2, 6) = 37.48, *p* = 0.0004; Lcn2: F (2, 6) = 42.18, *p* = 0.0003; pJAK2/JAK2: F (2, 6) = 29.96, *p* = 0.0008; pSTAT3/STAT3: F (2, 6) = 24.29, *p* = 0.0013).

## Discussion

In this study, we implanted previously developed implantable laser fibers [30] in SCI male rats and found that PBM alleviated secondary damage in the rats. Specifically, PBM led to outcomes including a moderate inflammatory response, a reduced level of tissue apoptosis, an increase in the number of surviving neurons, and improved motor function recovery. PBM is a reliable strategy for treating CNS diseases or injuries whose therapeutic effects have been validated in SCI models by different research teams [39–41]. As early as a decade ago, Byrnes et al. applied 810-nm low-level lasers for two consecutive weeks in SCI rats and found that PBM promoted neuronal regeneration and functional recovery [39]. Wu et al. also tested PBM with similar parameters in rats with SCI caused by two different types of trauma and again proved the repair-promoting effects of PBM [41]. Since then, increasing evidence has suggested that PBM primarily affects SCI models by inhibiting scar formation and regulating inflammation [12, 42, 43]. Our team has focused on optimizing PBM and exploring the underlying mechanism. Previous data have shown that PBM can facilitate alternatively activated macrophage/microglia polarization, relieve secondary neuroinflammation, and improve the ability of neurons to resist insults caused by trauma [15, 16, 44, 45]. However, in the corresponding studies, PBM was applied transcutaneously at the lesion site, and only a small dose of energy (approximately 6%) was able to penetrate tissue and reach the spinal cord surface, according to reports [39, 46]. In addition, due to the biphasic dose–response for PBM, simply increasing the irradiation power and exposure time may cause the optimal effect to be lost and may even cause tissue damage [47]. Therefore, we have developed biocompatible laser fibers that can be implanted beside the lamina in vivo to project laser energy directly onto the spinal cord surface with appropriate irradiation parameters. This strategy reduces energy scattering and passive absorption by body tissues, thus exerting a better biotherapeutic effect, and the feasibility and safety of this strategy has been reliably verified [30, 48].

Through this study, we found that inhibition of activated neurotoxic-type microglia and astrocytes is a potential mechanism of PBM. After SCI, microglia and astrocytes, as innate immune resident cells, begin to be activated and participate in secondary damage and tissue repair. Previous studies have classified activated microglia/macrophages and astrocytes simply as “good”/ “bad” types (M1/M2 microglia/macrophages or A1/A2 astrocytes) depending on their different roles in the pathological process. However, these simple binary divisions do not reflect the heterogeneity and plasticity of glial cells or the continuity of functional states [49, 50]. The activation states of microglia/macrophages and astrocytes are constantly changing under different disease or injury conditions. In the absence of better terminology, we considered a group of cells that expressed similar gene clusters as neurotoxic microglia and astrocytes (or proinflammatory microglia and astrocytes), while we considered cells that expressed another group of gene clusters neuroprotective (or anti-inflammatory). Here, we observed that the activation of microglia/macrophages and astrocytes occurred at different stages after SCI. Microglia/macrophages immediately responded to injury and began to activate, proliferate and translocate to the lesion area. The number of microglia/macrophages peaked at 7–14 dpi and then gradually subsided, while the activation of astrocytes was not obvious until 7 dpi and continued to strengthen within 28 dpi. PBM for two consecutive weeks significantly inhibited the activation of microglia/macrophages and astrocytes after SCI. The numbers of Iba1^+^ cells decreased within a certain range from the epicenter, although it was unclear whether this was due to PBM-mediated suppression of proliferation, recruitment, or both. It was clear, however, that the expression levels of iNOS, a classic marker of neurotoxic microglia/macrophages, were reduced in microglia/macrophages. Unlike in microglia/macrophages, PBM had little effect on astrocyte morphology, but it did downregulate the level of the neurotoxic astrocyte marker C3. Liddelow et al. reported that neurotoxic astrocytes are induced by C1q, IL-1α and TNF-α secreted by activated microglia [6], so we tested the levels of these three factors in injured spinal cord tissue at 3 dpi. The results indeed showed that PBM effectively downregulated the levels of these molecules, suggesting inhibition of astrocyte activation that was possibly followed by inhibition of microglial activation.

To further explore the activation mechanisms of neurotoxic microglia and astrocytes, we conducted RNA-seq experiments. We found that Lcn2 expression was strongly increased immediately after injury, which is consistent with other reports [22, 51]. Interestingly, the time course of JAK2-STAT3 signaling pathway activation after SCI was consistent with the changes in Lcn2 levels. According to other reports, JAK2-STAT3 pathway activation is necessary for neuroinflammation involving microglia and astrocytes, and inhibiting the JAK2-STAT3 pathway can reduce the reactivity of glial cells [52–54]. Lcn2 secreted by activated microglia and astrocytes under inflammatory conditions can recruit more inflammatory cells through JAK2-STAT3 pathway activation. This recruitment causes chemokines to be upregulated and secreted, thereby amplifying neuroinflammation and further enhancing the activation [17, 55, 56].

Subsequently, in vitro experiments were conducted to verify the roles of the Lcn2 and JAK2-STAT3 pathways in the activation of microglia and astrocytes. We first added a JAK2-STAT3 signaling pathway inhibitor (cucurbitacin I) to microglia and astrocytes. The results showed that cucurbitacin I effectively inhibited the upregulation of the JAK2-STAT3 pathway during the activation of microglia and astrocytes and simultaneously suppressed the upregulation of C3 and iNOS expression. Next, we separately transfected microglia and astrocytes with an adenovirus carrying shRNA in order to knock down Lcn2 and found that Lcn2 knockdown suppressed microglia/astrocyte activation and blocked JAK2-STAT3 pathway activation. Moreover, adding ReLcn2 protein to the culture medium of induced microglia and astrocytes further enhanced JAK2-STAT3 pathway activation and cell activation. However, ReLcn2 at certain concentrations did not significantly affect the Lcn2 and JAK2-STAT3 pathways in resting microglia and astrocytes and did not effectively promote the activation of microglia and astrocytes. This finding is in contrast to previous reports that elevated levels of ReLcn2 can induce microglia/astrocyte activation [19, 38, 57]. Therefore, we believe that a certain concentration of Lcn2 can stimulate the JAK2-STAT3 pathway and further accelerate cell activation only under conditions that induce reactivity in resting-state microglia and astrocytes. The Lcn2 and JAK2-STAT3 pathways jointly participate in the activation of microglia and astrocytes. Specifically, the two pathways engage in crosstalk for mutual regulation and can promote each other’s effects to enhance the degree of cell activation. Elucidation of the crosstalk of Lcn2/JAK2-STAT3 in the activation of microglia and astrocytes provides a better understanding of neuroinflammation. Blockade of the interaction may also partly explain the ability of PBM to ameliorate the neurotoxicity of microglia and astrocytes, which has been preliminarily validated in both in vivo and in vitro experiments.

PBM has been applied in experiments and in the clinic for more than 50 years. PBM seems to have a wide range of influences at the molecular, cellular and tissue levels, but the precise underlying biochemical mechanism has not yet been fully clarified, which has limited its widespread application. At present, the initial reaction is the absorption of photons by mitochondrial respiratory chain CCO, which then increases adenosine triphosphate (ATP) production, modulates reactive oxygen species (ROS), induces transcription factors, and causes downstream reactions [58]. For the first time, we propose that PBM inhibits the crosstalk of Lcn2/JAK2-STAT3 to suppress the activation of neurotoxic microglia and astrocytes, thereby alleviating neuroinflammation and facilitating tissue repair. Signaling associated with inflammation, such as mitogen-activated protein kinase (MAPK) signaling, nuclear factor kappa-Β (NF-κB) signaling, c-Jun N-terminal kinase (JNK) signaling, and Toll-like receptor (TLR)/myeloid differentiation primary response 88 (MYD88) pathway signaling, is suppressed by PBM; in contrast, the phosphatidylinositol 3-kinase (PI3K)/Akt pathways, extracellular signal-regulated kinase (ERK)/cyclic adenosine monophosphate-responsive element binding protein (CREB) pathways, and transforming growth factor-β (TGF-β)/Smad signaling pathways can be upregulated by PBM [59–63]. In cultured cells treated with PBM, the expression of GFAP and the secretion of cartilage sulfate proteoglycans (CSPGs) by primary astrocytes are decreased [16]; the levels of ROS and the axon retraction of dorsal root ganglion (DRG) neurons under oxidative stress conditions are improved [64]; NF-κB signaling in the activation of M1-type BMDM polarization is inhibited; and the protein kinase A (PKA)/CREB pathway participating in M2 polarization is activated [44, 45]. Our findings will contribute to clarification of the biochemical mechanism of PBM, especially since the roles of microglia and astrocytes in neuroinflammation have received increasing attention.

It is undeniable that we ignored the effect of sex on pathophysiology and recovery after SCI. Although more than 80% of patients are men aged approximately 30 years old [65], some reports have shown that female rodents may have better outcomes than male rodents after SCI [66, 67]. A recent study showed stronger total inflammation and more microglia at the lesion epicenter in SCI male rats than in SCI female rats, while the number of macrophages in the damaged area was higher in SCI female rats [68]. Considering that PBM in our study inhibited the activation of total Iba1^+^ cells indiscriminately, we predict that the therapeutic effects of PBM can be reproduced in female rat models of SCI without changing the overall conclusions, although this requires confirmation in the future.

## Conclusion

**Fig. 8:**
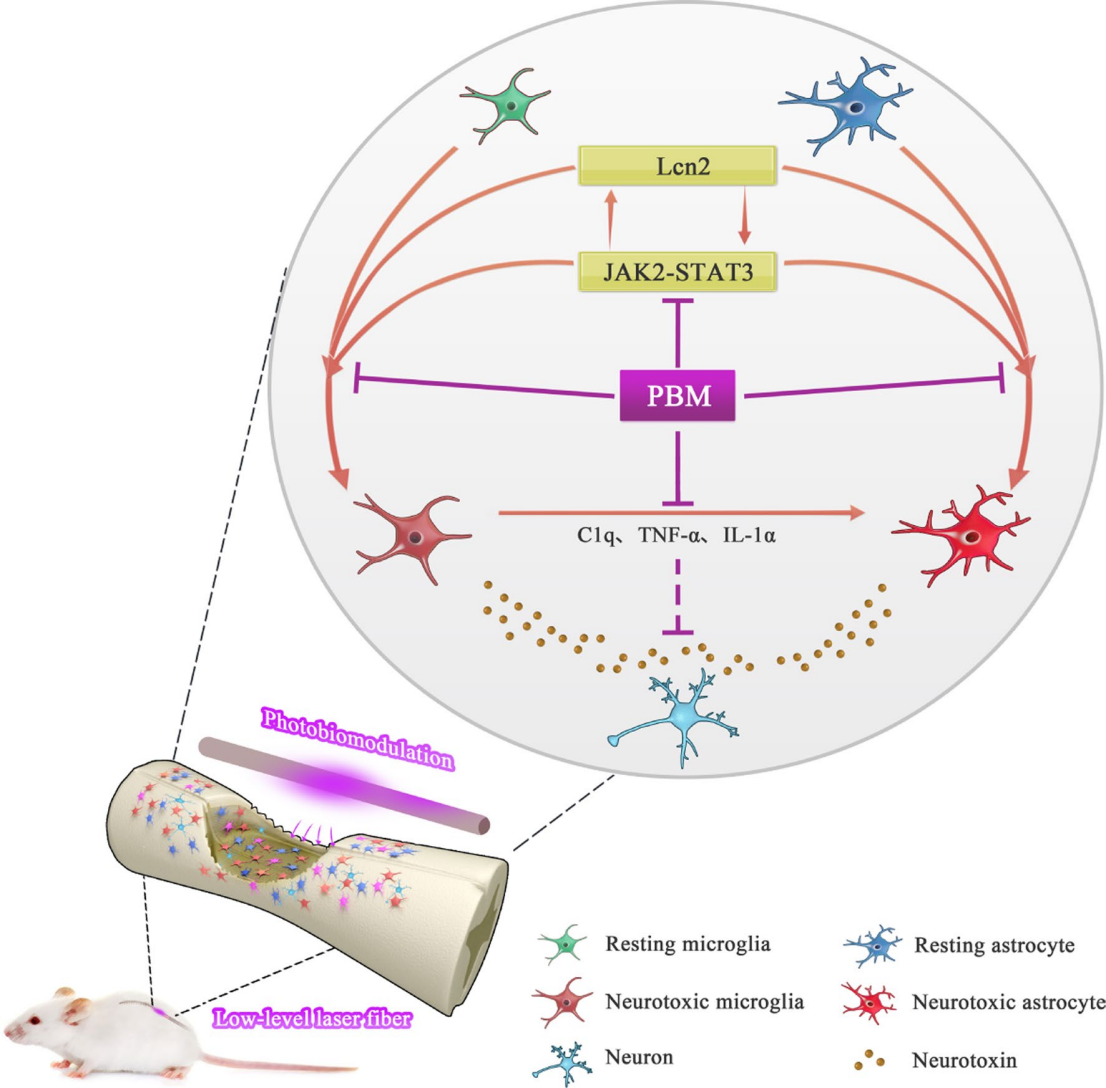
Schematic diagram showing the implantation of laser fibers in rats after SCI. Resting microglia and astrocytes begin to activate and participate in neuroinflammation after SCI. The activation of neurotoxic astrocytes is induced by the secretion of C1q, TNF-α, and IL-1α from activated microglia, and both neurotoxic microglia and neurotoxic astrocytes are harmful to neurons. The crosstalk of Lcn2/JAK2-STAT3 participates in the activation of microglia and astrocytes. Specifically, the JAK2-STAT3 pathway contributes to the activation of neurotoxic glial cells and the expression of Lcn2. In turn, Lcn2 exacerbates the activation of microglia and astrocytes and upregulates the activation of the JAK2-STAT3 pathway. The neuroprotective mechanism of PBM may be related to inhibition of the interaction of Lcn2 and the JAK2-STAT3 pathway, which is associated with neurotoxic microglia/astrocyte activation

In this study, we discovered the activation time course of neurotoxic microglia and astrocytes after SCI and proved that the crosstalk of Lcn2/JAK2-STAT3 is involved in this process. In addition, we verified that PBM can inhibit the activation of neurotoxic microglia and astrocytes by inhibiting Lcn2/JAK2-STAT3 crosstalk, thereby promoting recovery after SCI (Fig. 8).

## Data Availability

All raw data used in this manuscript are available on reasonable request.

## Abbreviations

PBM: Photobiomodulation
SCI: Spinal cord injury
CNS: Central nervous system
dpi: Days post-injury
GFAP: Glial fibrillary acidic protein
Iba1: Ionized calcium binding adapter molecule 1
JAK2-STAT3: Janus kinase 2-signal transducer and activator of transcription 3
Lcn2: Lipocalin 2
iNOS: Inducible nitric oxide synthase
TNF- α: Tumor necrosis factor-α
IL-1β: Interleukin-1β
IL-6: Interleukin-6
IL-1α: Interleukin-1α
C1q: Complement component 1q
BBB: Basso–Beattie–Bresnahan
LSS: Louisville Swim Scale
OCT: Optimal cutting temperature
PBS: Phosphate-buffered saline
DAPI: 4′,6-Diamidino-2-phenylindole
ROI: Region of interest
RT-PCR: Real-time quantitative PCR
ELISA: Enzyme-linked immunosorbent assay
RNA-seq: RNA sequencing
VSC4.1 cells: Ventral spinal cord 4.1 motoneurons
LPS: Lipopolysaccharide
IFN-γ: Interferon gamma
MOI: Multiplicity of infection
ReLcn2: Recombinant Lcn2 protein
PI: Propidium iodide
DMEM: Dulbecco’s modified Eagle medium
MCM (ACM): Microglia-conditioned medium (astrocyte-conditioned medium)
in-MG: Induced microglia
in-AST: Induced astrocyte
in-MCM (in-ACM): Induced microglia-conditioned medium (induced astrocyte-conditioned medium)
SD: Standard deviation
ANOVA: Analysis of variance
CCO: Cytochrome c oxidase
BMDMs: Bone marrow-derived macrophages
LLLT: Low-level laser therapy
